# Prevention of Tooth Discoloration Using Fluoride Varnish Immediately After Bleaching

**DOI:** 10.3390/jfb16070245

**Published:** 2025-07-03

**Authors:** Ryotaro Yago, Chiharu Kawamoto, Rafiqul Islam, Hirofumi Kaneko, Monica Yamauti, Masayuki Otsuki, Hidehiko Sano, Atsushi Tomokiyo

**Affiliations:** 1 Department of Restorative Dentistry, Graduate School of Dental Medicine, Hokkaido University, Kita 13 Nishi 7, Kita-ku, Sapporo 060-8586, Japan; 2 Department of Restorative Dentistry, Faculty of Dental Medicine, Hokkaido University, Kita 13 Nishi 7, Kita-ku, Sapporo 060-8586, Japan; 3 Department of Biomedical and Applied Science, Indiana University School of Dentistry, 1121 W. Michigan Street, Indianapolis, IN 46202, USA; 4 Cariology and Operative Dentistry, Department of Restorative Sciences, Graduate School of Medical and Dental Sciences, Institute of Science Tokyo, 1-5-45 Yushima, Bunkyo-ku, Tokyo 113-8549, Japan

**Keywords:** bovine tooth, fluoride varnish, restorative dentistry, tooth bleaching, tooth discoloration

## Abstract

Tooth bleaching is a widely used esthetic treatment; however, bleaching agents can temporarily alter the surface morphology of enamel, increasing surface roughness and porosity, which may lead to increased susceptibility to discoloration. This in vitro study investigated the effectiveness of fluoride varnish in preventing immediate discoloration of bovine incisors after bleaching. Specimens were bleached with 35% hydrogen peroxide and treated with either Clinpro White Varnish (CW) or Enamelast Fluoride Varnish (EN), whereas control specimens received no treatment after bleaching. The samples were immersed in coffee for 24 h, and the color difference (Δ*E*_00_) was calculated using the CIEDE2000 formula. The surface morphology of enamel was examined using SEM. The fluoride varnish groups showed significantly lower color difference values than the control group (*p* < 0.05), with Δ*E*_00_ reduced by approximately two-thirds in both the CW and EN groups. SEM observations showed that the enamel surfaces in the varnish-treated groups exhibited reduced surface irregularities compared to the untreated group, suggesting remineralization. These results suggest that the immediate application of fluoride varnish after bleaching can effectively reduce short-term discoloration by providing physical protection and promoting remineralization. Fluoride varnish may serve as a simple and effective strategy to maintain whitening outcomes and minimize early discoloration.

## 1. Introduction

In modern society, tooth whitening has become one of the most popular esthetic dental treatments, aimed at improving tooth color and overall smile appearance. According to clinical statistics, the demand for tooth whitening treatments has increased year by year, and there are currently a variety of tooth bleaching products available on the market for in-clinic or home use [1]. Tooth bleaching is widely accepted as an effective method for esthetic improvement and contributes to a beautiful smile [1,2]. Most of these bleaching agents use hydrogen peroxide or carbamide peroxide as the main ingredients, which achieve whitening effects by releasing oxygen free radicals and decomposing colored molecules in teeth [2].

Although bleaching can effectively improve the beauty of teeth in the short term, the durability of its effects is still a challenge. Studies demonstrated that color rebound after the bleaching procedure remains the crucial concern, and effective strategies are needed to ensure long-lasting results [3,4]. Change in tooth color after bleaching is caused by external staining agents such as coffee, wine, and tobacco. In addition, immediately after bleaching, demineralization of the enamel leads to changes in the microstructure of the tooth surface, making it more prone to staining by external factors [5,6,7,8,9,10]. It has been reported that the roughened enamel surface observed immediately after tooth bleaching is a temporary effect, and it can be remineralized by saliva [11,12]. Manufacturers recommend avoiding coffee, wine, and other substances known to cause discoloration for 24 h after bleaching due to the increased risk of discoloration [13,14]. Therefore, the whitening effect in the early stage after bleaching has become a clinical problem that needs to be addressed, and new approaches are required to maintain bleaching effects.

Traditionally, patients are advised to avoid drinking colored beverages or eating foods with strong pigments after bleaching, especially in the early stages [2]. However, compliance with these recommendations varies, and there is no guarantee that the tooth surface will not be completely stained. For this reason, we explored the use of materials with remineralization potential to protect the tooth surface structure and reduce the risk of early re-staining. Fluoride varnish was developed from the late 1960s to the early 1970s to prolong the contact time between fluoride and the tooth enamel [15]. This material has been shown to be effective in noninvasive dental treatment, primarily by promoting enamel remineralization, typically at a fluoride concentration of 22,600 ppm (5%) [16,17]. Fluoride varnish adheres to the tooth surface as a thin layer for an extended period, facilitating the deposition of calcium fluoride in enamel [18]. This precipitate acts as a fluoride reservoir, which is thought to promote the formation of fluorapatite [16]. These properties enable the use of fluoride varnish to prevent dental caries and to treat dentin hypersensitivity [19,20,21,22,23,24,25].

Currently, the commonly used fluoride varnish products in clinical practice include Clinpro White Varnish (CW) and Enamelast Fluoride Varnish (EN). CW is a fluoride varnish containing tricalcium phosphate [26]. It typically remains on tooth surfaces, forming a film that gradually wears off within 24 h. Sodium fluoride and tricalcium phosphate are dissolved and released as ions [27,28]. EN is a xylitol-containing fluoride varnish specifically formulated to enhance adhesion, thereby increasing its retention on the tooth surface and promoting fluoride release and uptake [29,30]. Although the role of fluoride varnish in enamel remineralization is well established, its potential as a preventive barrier against discoloration occurring shortly after bleaching has not been fully evaluated. We hypothesized that the application of a fluoride varnish—a long-lasting material—immediately after bleaching could modify the enamel surface through remineralization and inhibit discoloration. As the risk of discoloration is particularly high during the first 24 h after bleaching, immediate fluoride varnish application may represent an effective strategy to preserve whitening outcomes.

Therefore, this study aimed to assess the effectiveness of fluoride varnish in preventing discoloration immediately after bleaching. The null hypothesis was that there would be no significant difference in the color difference values in the fluoride varnish groups compared to the control group after 24 h.

## 2. Materials and Methods

### 2.1. Experimental Materials

The experimental materials used in this study were Clinpro White Varnish (CW; Solventum, Saint Paul, MN, USA) and Enamelast Fluoride Varnish (EN; Ultradent, South Jordan, UT, USA). The compositions of the tested materials are listed in Table 1.

### 2.2. Preparation of Specimens

**Figure 1:**
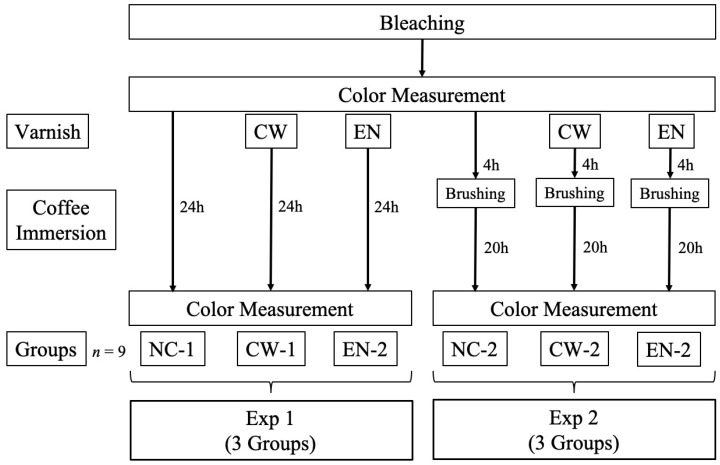
The experimental groups in this study. Abbreviation: NC, no varnish control; CW, Clinpro White Varnish; EN, Enamelast.

The required sample size was determined based on a power analysis using G*Power software (version 3.1, Heinrich-Heine-University Düsseldorf, Germany). Assuming a medium-to-large effect size (f = 0.4), an alpha level of 0.05, and a statistical power of 0.8, the minimum sample size required for one-way ANOVA with three groups was calculated to be 9 per group. Accordingly, a total of 54 bovine teeth, purchased from Yokohama City Shokuniku Kosha (Yokohama, Japan), were used to prepare enamel specimens for two independent experiments (Exp 1 and Exp 2), each consisting of three groups (*n* = 9). The specimens were randomly assigned to six groups in total, as illustrated in Figure 1.

**Figure 2:**
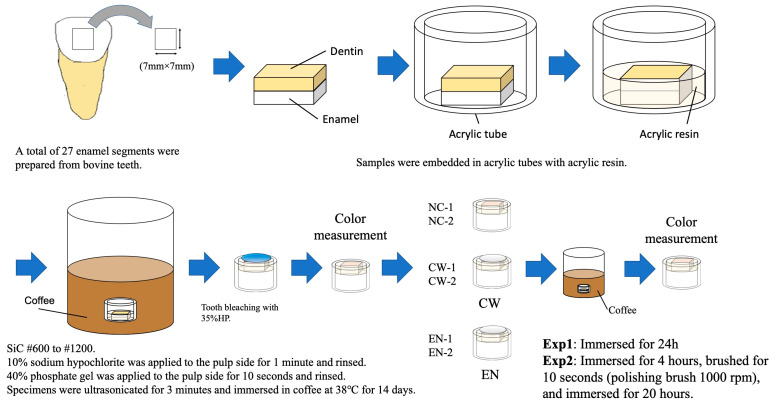
Sample preparation procedures for the specimens. Abbreviations: SiC, silicon carbide paper; HP, hydrogen peroxide; NC, no varnish control; CW, Clinpro White Varnish; EN, Enamelast Fluoride Varnish.

The specimen preparation procedure is illustrated in Figure 2. Bovine incisors were stored at −20 °C until use. Before specimen preparation, the teeth were thawed under running tap water at room temperature (23 °C) for 30 min. Each tooth was cleaned with a scalpel to remove soft tissue, and the roots were sectioned using a trimmer (MT-10 Model Trimmer, Morita, Tokyo, Japan). Square specimens measuring 7 mm × 7 mm × 2 mm (1 mm enamel and 1 mm dentin in thickness) were prepared using a diamond saw (Isomet; Buehler, Manassas, VA, USA). Each specimen was fixed in an acrylic tube (10 mm inner diameter and 10 mm height) using self-curing acrylic resin (Unifast II Clear, GC, Tokyo, Japan).

The enamel surfaces were manually polished under running water using silicon carbide paper (Sankyo-Rikagaku, Tokyo, Japan) with grit sizes of #600, #800, #1000, and #1200, for 30 s, followed by sonication in distilled water at room temperature for 3 min (FU-2H, Tokyo Garasu Kikai, Tokyo, Japan) to remove debris after the use of each grit. The process was the same for each grit, to standardize the surface. To remove residual organic material, 10% sodium hypochlorite solution was applied to the pulpal side for 1 min, followed by rinsing with distilled water for 30 s. This was followed by the application of 35% phosphoric acid gel (K-Etchant Gel; Kuraray Noritake, Tokyo, Japan) to the pulpal side for 10 s, then it was rinsed again with distilled water for 30 s. The specimens were finally sonicated in distilled water at room temperature for 5 min to remove debris. After preparation, the specimens were stored in distilled water until the experiment.

### 2.3. Color Measurement

The detailed procedure of color measurement after the sample preparation is illustrated in Figure 2. The specimens were immersed in coffee and stored at 37 °C for 14 d, with reference to previous studies [31,32]. The pulp side of each specimen was filled with Unifast II Clear, and the CIE L*a*b* values were measured at the center of the enamel surface using a spectrophotometer (CM-2600d, Konica Minolta, Tokyo, Japan) with a small-area view setting (SAV: φ3 mm/φ6 mm) under a D65 standard illuminant. Measurements were performed in a dark room against a black background.

A total of 54 specimens with L* values ranging from 30 to 45 were selected and divided into six groups (*n* = 9 each) based on the L* value. The groups were as follows: Groups (NC-1, NC-2)—no application after bleaching (control); Groups (CW-1, CW-2)—application of CW after bleaching; and Groups (EN-1, EN-2)—application of EN after bleaching.

Office bleaching was performed using 35% hydrogen peroxide (Hi-Lite; Shofu, Kyoto, Japan). According to the manufacturer’s instructions, the bleaching paste was mixed and applied to the enamel surface, left for 5 min, and then irradiated for 3 min using a light-curing device (Pencure 2000, Morita; irradiation intensity: 2000 mW/cm^2^), followed by a 1 min resting period. This cycle was repeated three times. After bleaching, CIE L*a*b* values were measured.

The bleached enamel surfaces were dried, and a thin layer of fluoride varnish was applied using an applicator brush. CW was applied to CW-1 and CW-2 and EN was applied to EN-1 and EN-2. The varnish was left undisturbed for 3 min. Subsequently, the specimens were immersed in coffee.

Two experimental protocols were used for each group.

Exp 1: Immersion in coffee for 24 h after bleaching.
Exp 2: Simulation of a clinical scenario in which patients brush their teeth after varnish application. The specimens were subsequently immersed in coffee for 24 h after bleaching. After 4 h of immersion, the samples were removed, brushed, and reimmersed. Brushing was performed for 10 s at 1000 rpm using a Robinson brush (Mersage brush, Shofu, Kyoto, Japan).

The specimens were rinsed with water, blot-dried, and subjected to color measurements. The color difference (Δ*E*_00_) was calculated based on the CIE L*a*b* values measured immediately after bleaching and after 24 h immersion in the staining solution using the CIEDE2000 color difference Formula (1), as described in our previous study [33].

(1) $$\Delta E_{00}={\left[{\left(\frac{{\Delta L}^{\prime }}{K_{L}S_{L}}\right)}^{2}+{\left(\frac{{\Delta C}^{\prime }}{K_{C}S_{C}}\right)}^{2}+{\left(\frac{{\Delta H}^{\prime }}{K_{H}S_{H}}\right)}^{2}+R_{T}\left(\frac{{\Delta C}^{\prime }}{K_{C}S_{C}}\right)\left(\frac{{\Delta H}^{\prime }}{K_{H}S_{H}}\right)\right]}^{1/2}$$

### 2.4. Scanning Electron Microscope (SEM) Observation

SEM specimens were prepared by cutting 3 mm × 3 mm sections from the central portion of bovine teeth. Each specimen was subjected to one of the following treatments: (a) no bleaching; (b) bleaching; (c) CW application after bleaching; (d) EN application after bleaching; (e) CW application after bleaching and brushing 4 h later; and (f) EN application after bleaching and brushing 4 h later. After gentle blot-drying using a Kimwipe, representative specimens from each group were randomly selected, and the specimens were sputter-coated with Pt-Pd for 120 s. The specimens were then mounted on aluminum stubs, and enamel surfaces were examined using a scanning electron microscope (SEM; S-4800, Hitachi, Tokyo, Japan). The entire surface of each specimen was first surveyed at low magnification, and representative areas showing characteristic features were selected for detailed imaging.

### 2.5. Statistical Analysis

Δ*E*_00_ values were analyzed using one-way analysis of variance (ANOVA), followed by Tukey’s honestly significant difference (HSD) test. All statistical analyses were performed using SPSS version 28 (IBM Corp., Armonk, NY, USA). Statistical significance was set at *p* < 0.05.

## 3. Results

### 3.1. Color Measurements

**Figure 3:**
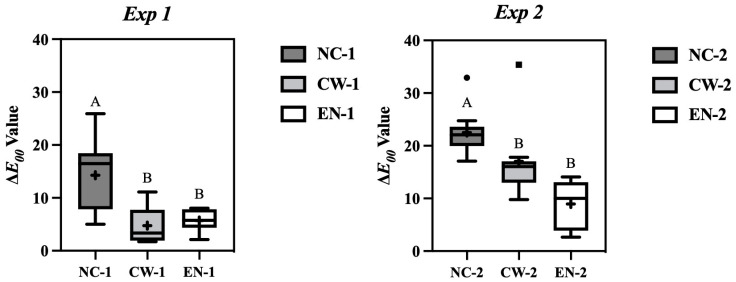
Δ*E*_00_ values after coffee immersion for 24 h under two conditions: Exp 1 (without brushing) and Exp 2 (with brushing 4 h after varnish application). NC-1/NC-2: no treatment after bleaching; CW-1/CW-2: application of Clinpro White Varnish; EN-1/EN-2: application of Enamelast. Groups sharing the same letter are not significantly different (*p* < 0.05). Lower Δ*E*_00_ values indicate better prevention of discoloration. + indicates the mean value. ● and ■ represent statistical outliers.

Figure 3 presents the results of the color measurement tests.

In Exp 1, the mean Δ*E*_00_ values for NC-1, CW-1, and EN-1 are 14.27, 4.74, and 5.70, respectively. A significant difference was observed between the intervention and the control groups. The Δ*E*_00_ values of the fluoride varnish groups immediately and 24 h after bleaching were approximately one-third of those in the control group.

In Exp 2, the mean Δ*E*_00_ values for NC-2, CW-2, and EN-2 are 22.51, 17.05, and 8.96, respectively. Again, a statistically significant difference was observed between the intervention and the control groups.

### 3.2. SEM Observations

**Figure 4:**
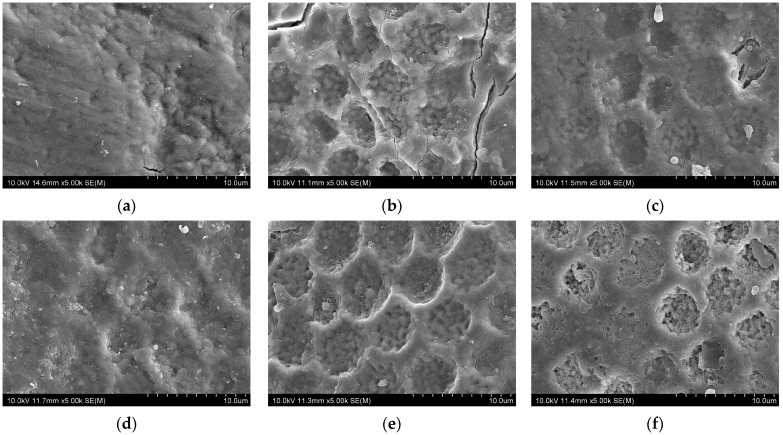
SEM images of enamel surfaces under different conditions. (**a**) No bleaching; (**b**) after bleaching; (**c**) CW application after bleaching; (**d**) EN application after bleaching; (**e**) CW application after bleaching and brushing after 4 h; (**f**) EN application after bleaching and brushing after 4 h.

Figure 4 presents SEM images of enamel surfaces under different conditions. After bleaching (Figure 4b), the enamel surface exhibited irregular pit morphology with increased roughness, indicating microstructural damage due to demineralization. In contrast, when fluoride varnish was applied immediately after bleaching (Figure 4c,d), surface irregularities were markedly reduced in both CW and EN groups. In the brushed groups (Figure 4e,f), the surface appearance resembled that of the bleached enamel (Figure 4b). However, in the EN group (Figure 4f), some pits remained partially occluded, suggesting the presence of residual varnish even after brushing.

**Figure 5:**
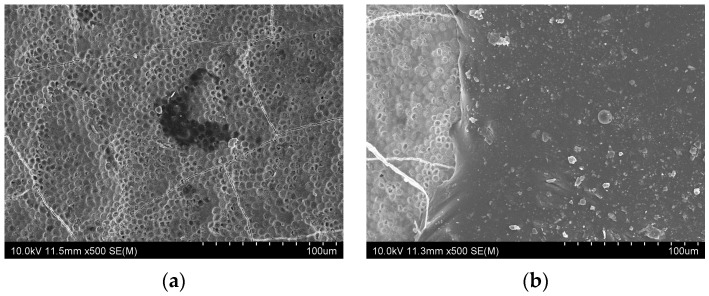
SEM images of fluoride varnish remnants on enamel surfaces after brushing. (**a**) CW application after bleaching and brushing after 4 h; (**b**) EN application after bleaching and brushing after 4 h.

Figure 5 presents images of the brushed samples. After CW application and brushing, no varnish residue was observed on the enamel surface (Figure 5a). In contrast, the EN-treated brushed sample exhibited visible remnants of a varnish layer (Figure 5b).

## 4. Discussion

The present study investigated whether applying fluoride varnish to bovine enamel immediately after bleaching could prevent discoloration caused by external pigments, as assessed by color difference after immersion in coffee for 24 h. Our findings showed that fluoride varnish application significantly reduced discoloration compared to the control group.

Discoloration occurring after bleaching is a common concern associated with esthetic tooth whitening procedures. Previous studies have suggested that the enamel surface becomes temporarily demineralized and morphologically altered after bleaching, increasing its susceptibility to external pigments such as those found in coffee and wine [5,6,7,8,9,10]. Despite the widespread use of fluoride varnish for remineralization [15,16,17,18], its potential to inhibit discoloration immediately after bleaching has not yet been thoroughly examined.

In this study, we evaluated the effectiveness of applying fluoride varnish immediately after bleaching to reduce early discoloration on bovine enamel surfaces. In addition to statistical significance, the clinical relevance of the observed Δ*E*_00_ values should be considered. A recent study reported that the clinical acceptability threshold for Δ*E*_00_ ranges from 1.80 to 2.84 [34]. In this study, although the Δ*E*_00_ values for all groups exceeded the clinical acceptability threshold, those in the varnish groups (particularly CW-1 and EN-1) were closest to this threshold, whereas the control group showed much higher values. The reduction in Δ*E*_00_ achieved by fluoride varnish application can be regarded as both statistically significant and clinically beneficial. A detailed analysis of the results suggests that the discoloration-inhibiting effect of the fluoride varnish is primarily attributable to two factors.

First, the physical coating effect of the varnish likely contributed to the inhibition of discoloration. Immediately after bleaching, the tooth surface becomes temporarily roughened and readily absorbs pigments [5,6,7,8,9,10]. Fluoride varnish is believed to form a protective layer on the enamel surface, thereby physically blocking pigment adhesion. This effect was particularly evident in Exp 1, where the fluoride varnish groups showed Δ*E*_00_ values approximately one-third of those in the control group. This indicates that fluoride varnish can reduce the risk of discoloration within 24 h of bleaching.

Second, the remineralization-promoting effect of fluoride varnish may have also contributed to the suppression of discoloration. After bleaching, the enamel undergoes changes resembling demineralization, resulting in microstructural alterations [7]. The presence of fluoride may promote remineralization, improve the surface integrity of the enamel, and reduce pigment adherence. In fact, previous studies have reported that enamel remineralization progresses following fluoride varnish application [35,36,37].

This remineralization process may involve not only the direct incorporation of fluoride into the enamel lattice to form fluorapatite, but also the deposition of calcium fluoride-like globules that function as reservoirs of fluoride ions [38,39,40,41]. These globules can gradually dissolve under acidic conditions, releasing fluoride to promote remineralization precisely when needed most, such as during dietary acid challenges [40,41]. The formation of fluorapatite has been reported to enhance the acid resistance of enamel [38]. This may help prevent the penetration of staining agents. Initial mineral deposition is also known to fill microdefects and surface porosities caused by bleaching [40,42], which may mechanically hinder pigment penetration. Therefore, the mechanism of discoloration prevention by fluoride varnish is thought to involve two main actions: enhancement of enamel remineralization and formation of a temporary surface barrier. These effects likely work together to inhibit the adhesion and penetration of staining agents during the initial period following bleaching. In addition, the temporary discoloration of teeth may occur after fluoride varnish is applied, usually lasting about 24 h until the outer layer of varnish is removed by brushing [15].

Under clinical conditions, the presence of saliva could substantially augment these processes [39,43]. Saliva supplies not only calcium and phosphate ions necessary for remineralization but also buffers pH fluctuations, creating a favorable environment for mineral deposition [38,40,43]. The ionic interplay between fluoride and salivary calcium/phosphate enhances the formation of stable mineral phases, accelerating surface repair [38,39,43]. Although our study was conducted in distilled water, the effects observed here may be underestimated compared to what might occur in vivo, where saliva’s dynamic flow and composition would provide additional protective and reparative benefits [38,40,43]. Future studies incorporating artificial saliva or dynamic pH cycling protocols would be valuable for validating these synergistic effects and for clarifying the kinetics and durability of fluoride varnish-induced remineralization.

Furthermore, it is important to consider that the rate and depth of remineralization can vary depending on the varnish formulation, the frequency of acid exposure, and individual variations in salivary composition [38,43]. The microlayer of calcium fluoride formed by varnish application may differ in morphology and stability between products, influencing the degree of long-term enamel protection [38,40]. Additionally, the potential for varnish components to interact with acquired enamel pellicle or biofilm could modulate fluoride bioavailability at the enamel surface [40,43]. These complex interactions highlight the need for comprehensive in situ or clinical studies that can capture the multifactorial nature of enamel remineralization in the oral environment.

Fluoride varnish may offer advantages over other fluoride-containing products, such as gels or mouth rinses, when used immediately after bleaching. While gels and rinses provide fluoride to promote remineralization, they generally require repeated applications and do not form a durable protective coating on the enamel surface [44,45]. In contrast, fluoride varnish can be applied quickly, forms a temporary protective layer, and simultaneously promotes remineralization through sustained fluoride release [18]. Furthermore, the high viscosity and adhesive properties of varnishes enable them to remain on the tooth surface for extended periods, even in the presence of saliva, thereby enhancing fluoride uptake by enamel [38,40]. Unlike gels and rinses, varnishes reduce dependence on patient compliance and can deliver a controlled, localized high concentration of fluoride at the site of application [38,43]. These features make fluoride varnish particularly valuable for mitigating sensitivity and supporting enamel protection in high-risk patients during the critical early period following bleaching.

Although this study focused on enamel surfaces, it is known that bleaching agents can also affect restorative materials, such as composite resins, potentially causing surface roughening and discoloration [46,47]. As many patients have such restorations, this is an important clinical consideration. Future studies should evaluate the protective effects of fluoride varnish or other preventive strategies on restorative materials subjected to bleaching.

The results of Exp 2 provide insights into the effect of tooth brushing on the discoloration-inhibitory properties of fluoride varnish. In this experiment, brushing with a Robinson brush was performed 4 h after bleaching, followed by 20 h of immersion in coffee. In NC-2, a marked increase in Δ*E*_00_ was observed, indicating continued discoloration, even after brushing. In contrast, the fluoride varnish groups exhibited suppressed increases in Δ*E*_00_, particularly in EN-2. This may be attributed to the adhesive and long-lasting properties of varnish. The adhesion-promoting formulation of EN likely enhances its retention on the enamel surface, maintaining its efficacy even after surface brushing [30]. In comparison, CW-2 exhibited a greater increase in Δ*E*_00_ compared to Exp 1, possibly because of partial removal of the varnish layer during brushing. Nevertheless, discoloration progression was still lower than in the control group, suggesting that CW also retained a certain degree of protective effect.

Based on the color difference measurements (Δ*E*_00_), both experiments demonstrated that discoloration was significantly reduced in the fluoride varnish groups compared to that in the control group. Thus, the null hypothesis, stating that there would be no significant difference in color differences among the groups after 24 h, was rejected.

In this study, SEM observations revealed that the enamel surface immediately after bleaching (Figure 4b) exhibited rough and irregular pit morphology, likely due to the demineralizing effect of hydrogen peroxide. In contrast, when fluoride varnish was applied immediately after bleaching (Figure 4c,d), surface irregularities were markedly reduced in both the CW and EN groups. The pits appeared shallower, and the overall surface showed reduced irregularity compared to the bleached enamel, suggesting that the varnish promoted remineralization [16].

The SEM observations revealed that varnish remnants remained after EN application, even after brushing. This finding likely reflects the high adhesion of EN owing to its synthetic resin matrix [29,30], allowing it to retain protective effects on the enamel surface. The compositional differences between CW and EN may also explain their differing performance. CW contains tricalcium phosphate, which provides calcium ions and may promote remineralization [26,27,28]; however, its retention appeared limited. In contrast, EN contains xylitol and exhibits greater adhesion, which, along with xylitol’s reported synergy with fluoride [48], may enhance remineralization and prolong its protective action against discoloration. In this study, SEM analysis was limited to qualitative observations to illustrate surface morphology differences among the groups. Quantitative image analysis, such as measuring pore depth or surface defect density, could provide greater objectivity and may help to further clarify the effects of bleaching and fluoride varnish application on enamel surfaces.

It has been reported that tooth bleaching roughens enamel surfaces and increase bacterial adhesion [49]. Fluoride varnish, a fluoride-releasing agent, promotes enamel remineralization, provides surface protection [18], and exhibits antimicrobial properties that inhibit bacterial metabolism and biofilm formation [50,51]. In addition, fluoride application is recommended after bleaching to reduce hypersensitivity, which is the most common side effect of bleaching [52,53]. Our findings support the multifaceted benefits of fluoride varnish. In addition to its established remineralizing and antibacterial properties, it also protects the roughened enamel surface from discoloration by acting as a physical barrier. Therefore, immediate application after bleaching may offer a simple and effective strategy to prevent early discoloration and to maintain dental health.

However, the effectiveness of fluoride varnishes on bleached tooth surfaces may vary depending on the product used. Although all materials in this study were used according to the manufacturer’s instructions, several limitations should be noted. The specimens were stored in distilled water, whereas artificial saliva may have provided a more clinically relevant environment by promoting remineralization. The static model lacked salivary flow or other dynamic oral factors. Moreover, the observation period was limited to 24 h, which does not reflect the long-term durability of fluoride varnish effects. In this study, coffee was used as the staining agent; however, different results might be observed with other discoloration factors, such as exposure to red wine or tobacco, or in the presence of pH cycling and artificial saliva. Future clinical studies under actual oral conditions will be important to validate the effectiveness of this approach.

In addition to its potential to prevent early discoloration after bleaching, the application of fluoride varnish may be integrated into post-bleaching care protocols in clinical settings. Such an approach could support not only esthetic outcomes by preserving the effects of bleaching but also contribute to the prevention of enamel sensitivity, a common side effect of tooth whitening procedures. Furthermore, fluoride varnish could play a role in comprehensive care strategies for patients with high risk of enamel erosion or structural defects following bleaching treatments. These possible applications highlight the value of incorporating fluoride varnish as a standard adjunct in esthetic dental procedures.

## 5. Conclusions

This in vitro study demonstrated that the application of fluoride varnish immediately after bleaching effectively reduced early discoloration on the enamel surfaces. Both CW and EN significantly decreased Δ*E*_00_ compared to the control group, with EN exhibiting greater retention and efficacy after brushing.

These results suggest that fluoride varnish provides a physical barrier against staining and may contribute to enamel surface protection. These findings highlight the potential of fluoride varnish application as a simple and effective strategy to maintain bleaching outcomes and to prevent short-term discoloration after bleaching.

## Figures and Tables

**Table 1:** Materials used in this study.

Material (Code)	ppm F	Composition	Percent per Weight	Manufacturer	Lot
Clinpro White Varnish (CW)	22,600	pentaerythritol glycerol ester of colophony resin	30–75	Solventum, Saint Paul, MN, USA	9660455
		n-hexane	10–15		
		ethyl alcohol	1–15		
		sodium fluoride	1–5		
		flavor enhancer	1–5		
		thickener	1–5		
		food-grade flavor	1–5		
		modified tricalcium phosphate	<5		
Enamelast Fluoride Varnish (EN)	22,600	synthetic resin	<50	Ultradent Products, South Jordan, UT, USA	BWB4Y
		ethyl alcohol	<15		
		sodium fluoride	<5		
		methyl ester of hydrogenated rosin	<5		
		citric acid	<3		
		xylitol	-		

“-”: not disclosed by the manufacturer.

## Data Availability

The original contributions of this study are included in the article.
